# Centralized Review of Alzheimer’s Disease and Related Dementias Biomedical Repositories and Computational Methods

**DOI:** 10.3390/bioengineering13060698

**Published:** 2026-06-18

**Authors:** Johaan Kathilankal Jis, Kewei Chen, Chen Zhao, Lingtao Chen, Seyedamin Pouriyeh, Zongxing Xie, Yixin Xie

**Affiliations:** 1 College of Computing and Software Engineering, Kennesaw State University, Marietta, GA 30060, USA; 2 College of Health Solutions, Arizona State University, Phoenix, AZ 85004, USA; 3 Bateman Physical Sciences Center, Arizona State University, Tempe, AZ 85281, USA

**Keywords:** Alzheimer’s disease, Alzheimer’s disease and related dementias, biomedical repositories, computational methods, machine learning, natural language processing, biomedical imaging, genomics, drug discovery, artificial intelligence

## Abstract

Alzheimer’s disease and related dementias (ADRD) are neurodegenerative conditions characterized by progressive cognitive and functional decline. AD pathology is associated with extracellular amyloid-β plaques, intracellular tau neurofibrillary tangles, synaptic dysfunction, and neuronal loss. AD accounts for approximately 60–80% of dementia cases globally. In 2022, AD was the seventh leading cause of death in the United States, and the number of Americans aged 65 and older living with Alzheimer’s dementia is projected to increase substantially by 2060. Despite decades of research, AD/ADRD data resources remain fragmented across clinical, imaging, genetic, genomic, and therapeutic domains. This paper addresses that gap by providing a centralized review of widely used AD/ADRD databases and computational methods. We first summarize computational approaches used to analyze these datasets, including machine learning (ML), natural language processing (NLP), and biomedical imaging. We then review eight databases classified into three categories: Clinical and Population Data, Genetics and Genomics, and Drug Discovery and Therapeutics. Finally, we discuss real-world applications, including early diagnosis, clinical decision support, personalized medicine, and drug-mechanism analysis. This review identifies opportunities for future work in data harmonization, cross-database compatibility, and robust, generalizable AI models for AD/ADRD research.

## 1. Introduction

**Figure 1:**
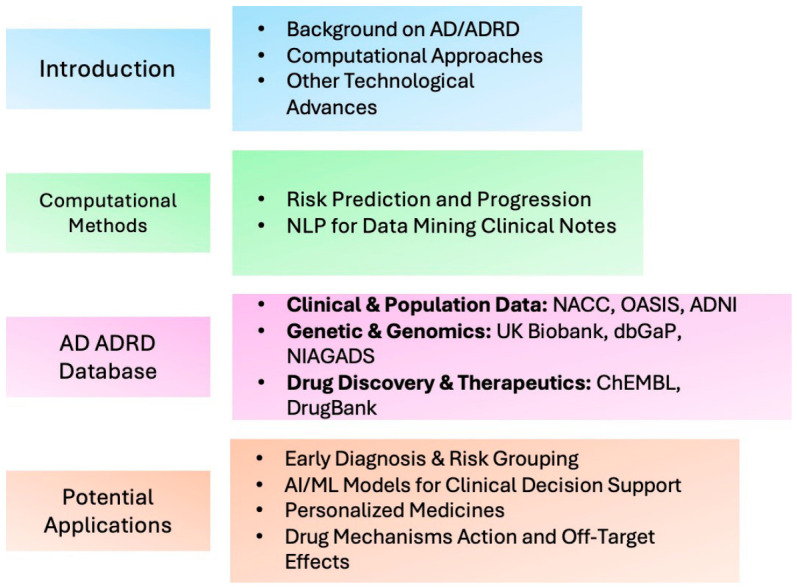
Flowchart of this paper, illustrating the subsections and their interconnections.

Alzheimer’s disease (AD) is a neurodegenerative disorder characterized by progressive cognitive and functional decline, with hallmark pathology including amyloid-β plaques and intracellular tau neurofibrillary tangles [1]. These pathological changes disrupt neural function and synaptic communication, contributing to widespread neuronal dysfunction and loss [2]. Over time, these processes are associated with memory loss, impaired daily functioning, and increased mortality [3]. AD is one of the most common causes of dementia, a broader clinical syndrome involving a decline in cognitive abilities. AD accounts for approximately 60–80% of dementia cases globally [4]. According to the Centers for Disease Control and Prevention (CDC), AD was the seventh leading cause of death in the United States in 2022 [5], making it a major concern for an aging population [6,7]. An estimated 7.2 million Americans age 65 and older are expected to have Alzheimer’s dementia by 2025, and projections suggest this number could nearly double to 13.8 million by 2060. The National Institute on Aging (NIA) has supported extensive AD research to identify disease-associated genetic alterations and molecular signatures in brain cell subtypes. However, the mechanisms linking molecular and genetic changes to brain-cell dysfunction remain incompletely understood. Ongoing research focuses on genetic variants such as APOE, multimodal biomarkers, and computational models for diagnosis and prognosis. Future AD/ADRD databases should integrate multimodal data sources, AI-ready pipelines, single-cell omics, advanced imaging, and high-throughput sequencing. Such integration can help researchers validate existing computational models and develop new methods tailored to the complexity of neurodegenerative disease. The organization of the paper is summarized in Figure 1.

The growing concern for AD led to the creation of different organizations and policies with the goal of raising awareness, advancing research, and helping individuals who have been affected by AD. The Division of Behavioral and Social Research (BSR) was established to support a broad range of research on aging, including studies that examine social and behavioral risk factors associated with AD [8]. In 2011, the National Alzheimer’s Project Act was signed into legislation to coordinate national support to accelerate AD/ADRD research and help provide better clinical care to individuals affected by this disease [9]. Since its discovery in the early 1980s, AD/ADRD databases have played an important role in bringing forth advancements to the field. These databases have helped researchers make important discoveries by understanding disease mechanisms [1,10], learning how biomarkers contribute to the degradation and development of disease, and developing new therapeutic practices [2]. However, the main problem with these databases is that while useful, there is no centralized resource that helps researchers identify and compare the range of available AD/ADRD databases based on their specific data needs, such as imaging, genetics, or clinical information. This paper aims to address that gap by summarizing and categorizing key databases relevant to AD/ADRD research.

To ensure comprehensive coverage while maintaining a focused review scope, we followed a four-step database-selection process. First, we searched the literature and database documentation through Google Scholar, PubMed, and IEEE Xplore using keywords such as “clinical ADRD data”, “Alzheimer’s Disease database”, “genomic AD databases”, and “AD datasets”. The PRISMA-style flow for database selection was as follows: 20 candidate databases or resources were identified, 12 were excluded because they were unavailable, outdated, inaccessible, duplicate resources, or outside the review scope, and eight databases were retained for detailed review. The retained databases were NACC, OASIS, ADNI, UK Biobank, dbGaP, NIAGADS, ChEMBL, and DrugBank. Thus, this review covers eight databases; no ninth database was retained after final screening. The selected databases were then cross-referenced with official database websites and NIH/NIA resources to verify accuracy.

## 2. Computational Methods for AD/ADRD

The growth of AD/ADRD databases has given researchers data resources for developing computational methods that advance disease understanding. Natural language processing (NLP), machine learning (ML), and biomedical imaging now support the extraction of clinically meaningful information from complex databases [11,12,13,14,15]. The following sections review how these technologies use AD/ADRD data sources to address current research and clinical challenges.

### 2.1. Machine Learning (ML)

Many ML techniques have shown promise for diagnosing AD [15,16]. Widely used classification algorithms include Support Vector Machines (SVM) [17], Artificial Neural Networks (ANN), Deep Learning (DL), Random Forests (RF), AdaBoost, K-Nearest Neighbors (KNN), and Logistic Regression (LR). SVMs identify separating hyperplanes and have been applied to diagnosis using brain volume and clinical features [18,19]. RF models aggregate ensembles of decision trees and are useful for high-dimensional or multimodal AD datasets [20,21]. AdaBoost combines weak learners through iterative reweighting and is often compared with RF and SVM in diagnostic studies [22]. KNN classifies new cases based on the most similar training examples and has been used in AD forecasting, MCI subtype classification, MRI-based feature studies, and multimodal ADNI analyses [23,24,25,26,27]. LR remains a common interpretable baseline for binary outcomes because it estimates how predictor variables contribute to a categorical dependent variable [28].

One of the trademarks of AD is the gradual loss of axons and neurons, causing loss of white matter integrity [29]. Although AD is mainly associated with changes in the brain, older individuals with conditions such as hypertension, hypercholesterolemia, diabetes, and cerebral small vessel disease (CSVD) often experience faster cognitive deterioration [30]. To differentiate AD-specific pathology, the National Institute of Aging and Alzheimer’s Association (NIAA-AA) proposed ATN frameworks that classified individuals based on biomarkers such as tau (T), amyloid beta (A), neurodegeneration (N), vascular contributions (V), and inflammation (I). Large-scale longitudinal studies like the Mayo Clinic Study of Aging [31], the Australian Imaging Biomarkers and Lifestyle (AIBL) Study of Aging [32], and the Alzheimer’s disease Neuro-imaging Initiative (ADNI) [33] study have collected biological, cognitive, neuroimaging, and lifestyle data. However, these resources present researchers with the ongoing challenge of identifying which subset of features is most important for understanding disease progression and early diagnosis of AD. Most studies have considered each individual measure from neuropsychological tests to be an independent variable or have limited analysis by controlling the number of outcomes per test. However, both these pathways often lead to the challenge of inflated false positives due to multiple comparisons. Consequently, many AD/ADRD pipelines incorporate feature selection and dimensionality reduction (e.g., filter-, wrapper-, and embedded-methods) to reduce redundancy, improve generalization, and maintain interpretability when working with high-dimensional clinical, imaging, and omics features.

Early detection of AD is important, especially as treatment options continue to evolve. Traditional assessments such as the Mini-Mental State Examination (MMSE) are useful, but they can be resource-intensive and may lack sensitivity to subtle cognitive changes in early AD [34]. Retinal imaging is an emerging complementary approach because retinal nerve fiber layer (RNFL) and ganglion cell-inner plexiform layer (GCIPL) changes have been reported in patients with MCI and AD [35,36,37]. Although ML, AI, and DL algorithms can extract information from these studies, model performance must still be evaluated across populations to ensure fairness and generalizability [38,39,40]. One recent study evaluated a deep-learning algorithm for MCI and AD dementia detection using Optical Coherence Tomography (OCT) scans across Asian and White cohorts [41]. The models used OCT-derived GCIPL and RNFL thickness maps generated with the Iowa Reference Algorithm [42]. The results suggest that OCT-based retinal features may help predict MCI and AD across ethnic groups, although retinal imaging remains an emerging approach and is not yet fully validated for routine AD diagnosis. Other work is exploring blood-based biomarkers as well as nasal and skin biomarkers as complementary, less invasive methods for early detection [43,44,45]. Antonioni et al. [46] reported that blood phosphorylated tau217 (pTau217) can differentiate amyloid-positive from amyloid-negative individuals across assay platforms, supporting its role as a scalable biomarker for early AD detection.

### 2.2. Behavioral and Cognitive Assessment Procedures

Behavioral testing in dementia studies combines brief screening, domain-specific neuropsychological tests, clinician-rated staging, and functional or neuropsychiatric symptom scales. Screening and staging tools commonly include the Mini-Mental State Examination (MMSE), Montreal Cognitive Assessment (MoCA), and Clinical Dementia Rating (CDR) or CDR Sum of Boxes (CDR-SB), which provide global measures of cognitive status and dementia severity [47,48,49]. Domain-specific tests are then used to identify patterns of impairment. Memory is often evaluated using word-list learning, delayed recall, or ADAS-Cog memory items. Executive function and processing speed are assessed with Trail Making, digit or number span, and similar attention tasks. Language is measured with naming tests such as the Multi-lingual Naming Test (MINT), Boston Naming Test, semantic fluency, and category fluency. Visuospatial function is commonly assessed using figure-copy or clock-drawing tasks. Behavioral and functional questionnaires, including Everyday Cognition (ECog), Functional Activities Questionnaire (FAQ), Neuropsychiatric Inventory (NPI/NPI-Q), Geriatric Depression Scale (GDS), and Activities of Daily Living scales, capture changes in mood, agitation, apathy, sleep, and other neuropsychiatric symptoms that may not be fully identified by cognitive test scores [50,51,52,53,54].

A standard behavioral-testing workflow begins with informed consent, demographic and medical history collection, and sensory/language screening to ensure that poor vision, literacy, hearing, or language mismatch does not influence performance. A trained examiner administers standardized instructions in a quiet setting, records raw responses, applies scoring rules, and notes factors such as medication use, fatigue, motor limitations, and caregiver assistance. Many dementia-related repositories also require an informant or study partner because neuropsychiatric symptoms and functional decline are often reported by caregivers. Test batteries or harmonized alternate forms are administered at repeated visits, allowing researchers to model practice effects, cognitive trajectories, and conversion from normal cognition to MCI or dementia. For computational studies, total scores, subscale scores, and derived composite scores can serve as model inputs, outcomes, or labels [55,56].

Software-supported behavioral testing is becoming more common. Examples include electronic data-capture systems for structured clinical forms, remote or web-based testing platforms used in large cohorts, and computerized batteries such as the NIH Toolbox Cognition Battery and the Cambridge Neuropsychological Test Automated Battery (CANTAB). These systems help reduce manual scoring errors, support remote screening, capture response time, and facilitate standardized data export for ML pipelines [57,58,59,60].

### 2.3. Natural Language Processing (NLP)

Pre-training has improved performance across many NLP tasks [61], including paraphrase detection [62] and natural language inference [63]. Two common strategies for adapting pre-trained language representations are fine-tuning and feature extraction. Fine-tuning, as in OpenAI’s Chat GPT [64], adds task-specific parameters and updates pre-trained parameters for downstream tasks. Feature-based models, such as ELMo [65], use task-specific architectures that incorporate pre-trained contextual representations as additional features. Earlier approaches often relied on unidirectional context, which can limit sentence-level performance and token-level tasks that require both left and right context.

Devlin et al. [66] proposed BERT: Bidirectional Encoder Representations from Transformers. BERT uses a masked language model (MLM), which randomly masks input tokens and predicts the original vocabulary identifiers from context. BERT uses a multi-layer bidirectional Transformer architecture introduced by Vaswani et al. [67]. Instead of training only left-to-right or right-to-left, BERT uses two unsupervised objectives: masked LM and next sentence prediction (NSP). Masked LM is related to the cloze procedure [68], while NSP helps the model learn relationships between sentence pairs [66]. Because BERT is trained on general-domain corpora such as BooksCorpus and Wikipedia, it may struggle with specialized abbreviations, terminology, and clinical context. Domain-specific BERT models such as ClinicalBERT and BlueBERT address this limitation by adapting transformer models to medical and biomedical vocabulary.

ClinicalBERT’s goal is to model clinical notes so that clinical insights and medical predictions can be derived from them [69]. ClinicalBERT is trained on clinical notes and discharge summaries from the Medical Information Mart for Intensive Care III (MIMIC-III), which contain information useful for disease prediction, treatment-outcome relationships, and corpus summarization. These clinical data are converted into numerical representations using token embeddings, segment embeddings, and position embeddings. These embeddings are added together to form the final input representation for each token, along with a special classification token (CLS) at the beginning of each sequence for classification tasks.

BlueBERT or Biomedical Language Understanding Evaluation (BLUE) benchmark is another variant of BERT that was developed to promote research in language representation within the domain of biomedicine. What sets BlueBERT different is that its BERT models cover a broad range of clinical and biomedical documents, support tasks beyond sentence-pair classification, and provide a complete codebase that allows researchers to rebuild datasets without losing any data instances [70]. Other domain-specific BERT variants are also available, such as BioBERT [71] and AD-BERT [72], both of which extend BERT’s capabilities to biomedical domains.

### 2.4. Biomedical Image Processing

While many different types of neural network architectures exist today, each has its own strengths and weaknesses. Convolutional architectures stand out as the key enabler for computer vision [73,74]. To better help with diagnosing using biomedical imaging, researchers will have to adopt a multimodal approach to analyze neuroimaging data. Magnetic Resonance Imaging (MRI) provides structural information, and T-1 weighted (T1-w) gives important data crucial for tracking atrophy and visualizing brain anatomy. T2-weighted (T2) images are another modality that is primarily used to identify pathologies like inflammation, demyelination, and white matter hyperintensities. Diffusion Tensor Imaging (DTI) provides researchers with useful data on the brain’s structural connectivity. Another imaging modality is Positron Emission Tomography (PET), which offers molecular imaging using radioactive tracers and more specific tau-PET tracers, which can help visualize tau tangles within the brain. Tau-PET imaging has been shown to correlate with neuronal injury and cognitive decline in Alzheimer’s disease, and is increasingly used for staging tau pathology [75,76].

Several studies have combined CNN-like architectures with self-attention [77], while others have proposed transformer-based alternatives [78]. The Vision Transformer (ViT) applies a standard Transformer architecture directly to images by splitting an image into patches, mapping those patches to linear embeddings, and treating the embeddings as token sequences [79]. ViT models can perform well when trained at scale, but they may generalize poorly on smaller datasets without adequate regularization because they lack some of the inductive biases built into CNN architectures.

Although CNNs have shown promise for medical-image analysis, neuroimaging-based diagnosis remains challenging when multimodal data are incomplete [80,81,82]. Compared with traditional CNNs, deep CNNs offer greater modeling capacity and depth for capturing nonlinear relationships [82,83]. Li et al. [84] addressed missing multimodal neuroimaging data with a 3D CNN architecture that learns to generate one imaging modality from another. In an ADNI-based study, the dataset included 830 subjects: 198 AD patients, 229 healthy controls, and 403 MCI patients, including 236 with stable MCI. T1-weighted MRI scans were preprocessed using skull stripping, cerebellum removal, and intensity inhomogeneity correction [85,86]. PET scans were then registered to corresponding MRI scans [87].

Functional MRI (fMRI) supplements structural MRI studies by measuring blood oxygen level-dependent (BOLD) signal fluctuations related to resting-state functional connectivity or task-evoked activity. Because resting-state fMRI does not require patients to perform demanding cognitive tasks during scanning, it can be useful for participants with memory loss, impaired executive function, or reduced attention. Commonly extracted features include default mode network (DMN) connectivity, hippocampal and posterior-cingulate connectivity, precuneus connectivity, frontoparietal and executive-control network connectivity, salience network connectivity, regional homogeneity, low-frequency fluctuation measures, graph-theoretic network measures, and dynamic functional connectivity. These features can reveal insights into cognitive dysfunction related to attention, executive function, visuospatial processing, and episodic memory [88,89,90,91].

CNNs can be implemented at several stages of the fMRI analysis pipeline. They can learn spatial patterns directly from 4D or 3D BOLD images, where convolutional filters capture network-level and local activation patterns. CNNs can also be applied to functional-connectivity matrices by treating each matrix as an image whose entries represent pairwise synchronization between brain regions. Hybrid CNN-recurrent architectures can analyze dynamic functional connectivity by combining convolutional layers for spatial feature extraction with recurrent or long short-term memory layers for temporal patterns across scanning windows. Although these methods have been used to classify cognitively normal controls, fMRI-based CNN models require careful preprocessing, temporal filtering, motion correction, and external validation because BOLD signals are sensitive to head motion, physiological noise, and preprocessing-pipeline variation [92,93,94].

## 3. AD/ADRD Databases

In this section, we review eight widely used AD/ADRD databases. For each, we provide a concise overview highlighting its origins, data types, accessibility, and applications. This structured presentation is intended to help readers quickly identify which database best aligns with their research needs without requiring them to sift through extensive documentation. This review focuses on eight databases classified into three categories: Clinical and Population Data, Genomics and Genetics, and Drug Discovery and Therapeutics.

### 3.1. Clinical and Population Data

Clinical Data is the type of data that is collected throughout a patient’s healthcare journey [95]. It includes everything from their laboratory results, medical history, doctor visits, medications, treatment plans, etc. Structured data for this category of data includes vital signs, test results, etc. It is mainly used for generating epidemiological trends, statistical insights, and monitoring patient health over their lifetime [96]. Unstructured data contains data that contains free-text entries and narrative descriptions, which are commonly found in clinical notes, detailed image reports, and medical records [97,98]. This kind of data mostly lacks standardization, presenting a challenge in creating an automated solution for diagnosing AD. The successful extraction of detailed, quantitative risk data can often be used in applications of advanced technologies like NLP [99,100]. While structured data can be used for quantitative analysis, it often lacks the microscopic detail and context that is often found within narrative notes made about the patient [101,102]. On the other hand, unstructured data holds great qualitative value as it captures a physician’s observations and a patient’s symptom description; processing this type of data at scale is computationally heavy. In today’s healthcare landscape, clinical data serves as the foundation for evidence-based medicine practices. It encourages professionals to make informed decisions on patients’ treatment plans, diagnosis, and to calculate future outcomes from treatment. Beyond patient care, clinical data also plays a role in medical research by identifying critical risk factors, evaluating the effectiveness of treatment outcomes, and helping in the creation of new clinical protocols. Population Data is another type of data source that gives researchers a statistical overview of a group or category of individuals, which forms the basis for understanding societal trends, guided policymaking, and guided research [103]. Population Data is defined as a collection of individuals who share a set of characteristics. Most of the time, Population Data are defined by geographical boundaries, such as “people of Georgia” or “people of the United States”. However, the definitions extend beyond geographical boundaries since most Population Data studies focus on understanding similarities between people. This type of data is important for answering questions such as “Which populations are at higher genetic risk for developing Alzheimer’s disease?”, “How does access to healthcare and early diagnosis vary among different demographic groups?”, etc.

#### 3.1.1. National Alzheimer’s Coordinating Center (NACC)

The NACC [104] was established by the National Institute on Aging (NIA) in 1999. It serves as the central hub for data collection, communication, and collaboration across NIA’s Alzheimer’s Disease Research Centers (ADRC) Program [105]. Over more than 25 years, NACC has worked with 42 former and current ADRCs across the United States to build one of the world’s largest and most comprehensive datasets for AD and ADRD [106,107]. The NACC Data Platform contains standardized multimodal data on more than 50,000 participants, ranging from cognitively normal individuals to those with mild cognitive impairment or dementia [108]. These multimodal datasets include clinical assessments, neuroimaging, genetic data, and other data types that enable comprehensive cross-domain analysis. For behavioral and cognitive data, NACC relies on the Uniform Data Set (UDS), which includes clinician-rated dementia staging, informant-based functional and neuropsychiatric forms, and a standardized neuropsychological battery. UDS versions include tools such as the CDR/CDR-SB, FAQ, NPI-Q, GDS, MoCA or MMSE depending on the UDS version, Craft Story recall, Benson Complex Figure, Number Span, MINT, cate-gory fluency, and Trail Making tests, allowing researchers to model memory, executive function, language, visuospatial ability, mood, daily functioning, and behavioral symptoms [55,107,108,109]. Since 2005, ADRCs have contributed to the UDS through longitudinal, prospective, and standardized clinical evaluations of participants in the NIA ADRC program [55]. During each participant’s annual UDS visit, clinicians collect approximately 18 forms covering topics from demographics to diagnosis. The UDS represents total enrollment from the ADRC program since 2005 and includes individuals with dementia as well as those without dementia. Each ADRC admits participants according to its own recruitment procedures, such as self-referrals, outreach through community organizations, or referrals from clinicians. Therefore, the NACC population is not a representative sample of the United States population [110]. NACC demographics have historically been skewed toward non-Hispanic White participants, although recent efforts are expanding representation of underrepresented populations.

ADRCs also contribute to the Neuropathology Data Set (NPDS), which contains post-mortem data. It also contains details about pathological staging systems, like the Thal phase (A Score) [111], Braak neurofibrillary stage (B score) [112], and Neuritic plaque score (C score) [113], along with their derived scores like Alzheimer’s disease neuro-pathology change (ADNC score) [114], and Alzheimer’s disease neuro-pathology present (B+C Score). The Neuropathology Protocol Procedures Survey (NPPS) dataset, along with the available data, describes the methods that could be employed by each center for preparation, examination, storage, and specimen acquisition.

Other forms of data that stores NACC in its datasets are imaging data, which consists of MRI and PET scans. These data follow the standardized centralized Alzheimer’s Neuroimaging (SCAN) protocols to ensure comparability across different ADRCs. SCAN includes PET and MRI analysis results, including cortical thickness, Standardized Uptake Value Ratios (SUVRs), surface area, along with quality control and numerical summary information [115]. While SUVRs provide important quantitative measures, current research is underway to harmonize these PET measures by converting them into Centiloid units, enabling standardized comparison between acquisition protocols and different tracers. Researchers can also request defaced PET and MRI images, which are made available through partners like the Laboratory of Neuro Imaging (LONI) [116]. While NACC also collects and shares “Mixed Protocol” data, which includes MRI and PET scans acquired before January 2021 or outside standardized protocols. These heterogeneous datasets are in the process of being systematically labeled and cleaned and are being added to the database as standardized, analysis-ready data.

The NACC database also serves as a hub for connecting neuropathological and clinical data with genetic and bio-specimen information. This was made possible through a partnership with key partners, including the National Institute on Aging Genetics of Alzheimer’s Disease Data Storage Site (NIAGADS), the National Cell Repository for Alzheimer’s Disease (NCARD), and Alzheimer’s Disease Genetic Consortium (ADGC). From NCARD, NACC integrates fluid biospecimen metadata and analysis data, which includes CSF biomarkers (A-beta, p-tau, t-tau) and blood-based biomarkers. Genomics and Genetics data, including the Apolipoprotein E (APOE) gene, AD mutations (e.g., PS1, PS2, APP), and well-known Frontotemporal Lobar Degeneration (FTLD) mutations (e.g., PGRN, FUS, MAPT), are connected to NACC IDs even though their primary genomic data is found with NACC’s partners like ADGC and NIAGADS [117].

The underlying architecture of NACC was initially developed using Statistical Analysis System (SAS) products along with its own web-based data management software to control and support data entry. The NACC database consists of over 900 data elements, each of which is grouped logically into individual datasets whose primary function is to organize, simplify collection, and documentation. The NACC database is a relational database that allows different datasets to be connected through shared keys like “patient ID” and “center ID”. Each “subject ID” is assigned by their contributing center to identify participants across datasets. This allows for a cohesive system in which different types of information for a single individual can be linked and analyzed together. To ensure consistency of protocols across ADRCs, NACC also provides a suite of documentation tools, which includes detailed coding guidebooks, UDS Forms packets, and Data Element Dictionaries (DED), all of which are useful for guiding clinicians on how to interpret data correctly. For researchers, the UDS Researchers Data Dictionary gives a clear framework to analyze demographic and clinical variables to ensure comparability and quality across the data network [106].

Once all of the data is collected at the respective ADRCs, their data cores process information before being submitted to NACC. Before submission, the data core at each ADRC prepares and processes the collected information. To do this, NACC offers multiple submission methods that are tailored to each of the centers. One of such methods is Batch File Upload, which allows for ADRCs to submit large volumes of data in CSV through the ADRC portal. The most advanced method is the Programmatic Upload via. Application Programming Interface (API) [118]. This allows for ADRCs to build with direct integration between their local systems and the NACC data platform for automated data transmission. Another aspect of the submission process is the requirement of National Alzheimer’s Coordinating Center Identifiers (NACCIDs) that are assigned at the time of participant consent. NACCIDs ensure the ability to link multimodal datatypes across partner repositories like NIAGADS, NCARD, and NACC’s own repositories.

When data reaches NACC, a robust multi-layered data validation pipeline ensures that data entering the NACC database meets quality standards. The validation pipeline starts with CSV Screening, which is the initial stage that checks if the submitted CSV file follows the correct naming conventions. After CSV Screening, the file goes to CSV Format check, which verifies that the submitted CSV has the correct data types and headers. It also makes sure that the CSV only includes NACC-accepted variables for the respective modules and matches the NACC Data Element Dictionary (DED). Next, the file goes into the Identifier lookup stage, in which for each row in the CSV file, the pipeline tries to match the Patient ID (PTID) and ADRC’s internal ID (ADCID) to an existing NACCID. If a matching NACCID is not found, the record is not processed further. The records that pass the Identifier Lookup stage undergo important data transformations that are specific to the submitted module, which is followed by pre-processing checks. Finally, the record goes into NACC-published data for quality checks, and any rows that fail these checks are rejected. Errors that are reported during these automation checks are reported to the submitting ADRC, and some common issues are generated as “alerts” and are sent for manual approval and inspection before the submission can be finalized. To ensure data quality, NACC provides the Form Validator package, which allows developers at ADRCs to run error checks and validate data on NACC-specific forms before submission to help centers identify and resolve errors before submission to speed up the error-checking process and finalize the process at the NACC level.

Access to NACC data is supported through various tools. The Quick-Access File Data Request System (QAF-DRS) makes researchers complete a Data Use Agreement (DUA) [119] and submit a data request. Once approved, data is released within 48 h. The Web-based Query System allows its users to perform initial searches on the database to determine whether NACC holds a sufficient number of subjects for their individual research. This includes various variables such as sex, race, primary diagnosis, age at visit, etc. It is important to note that the web-based query system can only be used for planning and cannot be used for publication, as it needs to be followed up with a different procedure. A more advanced tool to access data is the Multimodal Query Tool (MQT). MQT allows for real-time search visualization and helps build cohorts across different data modalities. Researchers can refine query criteria, export NACCIDs to retrieve extra data from NACC and its partners, and monitor sample sizes. MQT v1 is currently a prototype and is in the validation stage. For users who are approved at pilot project partner sites, NACC offers access through LINKAGE Enclave, through which researchers can analyze integrated datasets that combine datasets, such as Medicare and Medicaid claims, UDS with EHR, etc.

NACC uses REDCap for its data collection and storage. REDCap technology is Health Insurance Portability and Accountability Act (HIPAA) compliant, ensuring that all participant data is maintained in accordance with federal privacy regulations [120]. Once researchers are granted access to their requested NACC data, NACC offers guidance to help with data interpretation and analysis. One of the key resources to help with this is the NACC Handbook, which gives a detailed overview of the database and data summary tables that provide participant counts by diagnosis and demographics. The handbook offers to help researchers navigate the dataset and apply analytical methods to it. These include, but are not limited to, processes like merging datasets using NACCID, restricting analyses to specific visits, and filtering cohorts based on particular UDS form versions or the number of visits. NACC additionally provides recommendations on selecting relevant biomarker and imaging data to include in analyses. Over the years, the NACC database has become one of the foundations of global AD/ADRD research [121]. In the past 3 years, NACC has observed a 230% increase in monthly data requests as well as 26% rise in publications.

Beyond its purpose as a data platform, NACC is also active in investigator development and research funding. Since 2000, NACC has funded investigator-initiated and collaborative research projects at NIA-funded ADRCs [106]. These have enabled research in diverse topics like genetic risk modeling, inflammatory pathways, neuroimaging-based prognostics, mixed neuropathologies, and sensory dysfunction in AD. Studies using NACC data have directly made updates to the NIA-AA Research Framework, which enables characterization of developing neuropathological conditions like limbic-predominant age-related TDP-43 encephalopathy (LATE) [122], primary age-related tauopathy (PART) [123]. Due to the multimodal nature of NACC datasets, research has deepened the understanding of AD/ADRD progression and pathogenesis. Large-scale studies involving these data have explained disease patterns and the impact of coexisting conditions like protective or risk genetic factors and brain injury.

#### 3.1.2. Open Access Series of Imaging Studies (OASIS) Database

The Open Access Series of Imaging Studies (OASIS) [124] is a seminal project whose ultimate aim is to make brain neuroimaging datasets freely available to the global scientific community [125]. Its mission is to foster future discoveries in clinical and basic neuroscience, particularly in AD/ADRD research. OASIS began as a collaborative effort among leading research institutions. It was first developed at the Washington University ADRC with contributions from Dr. Randy Buckner’s lab and the Neuroinformatics Research Group (NRG) at Washington University School of Medicine.

The OASIS database consists of four datasets, and its reliability depends on standardized procedures and image screening for processing or acquisition errors. These procedures help ensure viable data, while the inclusion of images with typical MR acquisition artifacts allows researchers to test the robustness of analytical techniques across different brain-imaging conditions [126]. Although each OASIS dataset has distinct imaging, clinical, and cognitive data tailored to specific research goals, the datasets share a common data-sharing infrastructure. OASIS is therefore a multimodal resource that combines functional and structural imaging with clinical evaluations and cognitive scores across cohorts. To promote ease of use, OASIS data are provided in industry-accepted formats such as the Brain Imaging Data Structure (BIDS) [127], which uses NIFTI files with corresponding JSON metadata. BIDS organizes neuroimaging data in a consistent file hierarchy, making datasets easier to analyze and process.

OASIS-1 was the foundational dataset within the OASIS collection, which was designed to provide a sample of brain imaging data across the adult lifespan to help researchers to study age-related changes and early markers of neurodegenerative disease. OASIS-1 consists of 416 subjects whose age range from 18 to 96 years [128]. Among the 416 participants, 100 subjects over the age of 60 were clinically diagnosed with moderate to mild AD. The primary format in which image data is stored in OASIS-1 is through T1-weighted MRI [129]. For each individual, 3 or 4 T1-weighted MRI scans were collected during a single scanning session using a 1.5-T Vision scanner, to ensure that the data is of high quality and can be used for a wide range of advanced computational analysis and the development of segmentation algorithms.

Building on OASIS-1, OASIS-2 was developed to help researchers track brain changes and cognitive decline over time in the same cohort. OASIS-2 contains a longitudinal collection of 150 subjects aged 60 to 96 years [130]. Each participant was scanned during two or more visits, with at least one year between visits. Of the participants, 72 remained non-demented throughout the study, while 64 were classified as demented at their initial visits and remained demented [131]. Among the 64 demented participants, 51 were diagnosed with mild to moderate AD, and 14 participants transitioned from a non-demented state to dementia during later visits. Like OASIS-1, OASIS-2 stores longitudinal T1-weighted structural MRI for each subject but does not provide the resting-state fMRI coverage later introduced in OASIS-3.

After OASIS-2, OASIS-3 made a leap forward in integrating diverse clinical, imaging, and cognitive data collected over an extensive period of time. OASIS-3 is a multimodal dataset consisting of a wide range of clinical data and image sequences [132]. From MR, OASIS-3 includes T1-weighted (T1w), T2-weighted (T2w), Arterial Spin Labeling (ASL) [133], Time Of Flight (TOF), Fluid-Attenuated Inversion Recovery (FLAIR), Resting-state Blood Oxygen Level Dependent (BOLD), Diffusion Tensor Imaging (DTI) sequences, Fluid-Attenuated Inversion Recovery (FLAIR) [134], and Susceptibility Weighted Imaging (SWI) [135]. These data allow researchers to better investigate white matter pathways, cerebral blood flow, functional connectivity, and structural integrity. In addition to MRI data, OASIS-3 also includes PET imaging data from different tracers like Florbetapir (AV45), Fluorodeoxyglucose (FDG), and Pittsburgh Compound B (PIB), giving researchers insights into metabolic activity and amyloid plaque burden [136]. A sub-project under OASIS-3 AV1451 provides users with 451 Tau PET sessions and post-processed PET Unified Pipeline (PUP) data [137]. Beyond imaging data, OASIS-3 includes a vast collection of cognitive and clinical data, which are standardized through UDS, which cover a broad spectrum of details like detailed medical history, neurological evaluations, physical examinations, etc. [91].

The latest addition to the OASIS collection is OASIS-4, which focuses on a clinical cohort evaluated for dementia and memory disorders [124,138]. OASIS-4 includes 663 subjects aged 21 to 94 years. Participants reported symptoms related to AD/ADRD, making the dataset useful for studying early symptoms and diagnosis. OASIS-4 includes neuropsychometric evaluations, neuroimaging assessments, clinical assessments, and CSF analyses [139]. OASIS-4 is a separate dataset, not an update to the OASIS-3 Longitudinal Multimodal Neuroimaging dataset.

One foundation of OASIS is the anonymization and de-identification of participant data. Distributed data are assigned random IDs, and identifying information is removed from image files [126]. Connections between random IDs and original identifiers are destroyed before release to protect participant privacy. Some datasets may include non-defaced structural images, but these are safeguarded by a Data Use Agreement (DUA) that prohibits re-identification attempts and requires acknowledgement before results are disseminated. The core Archive Toolkit, XNAT, is an open-science platform for processing, archiving, secure distribution, and data management [140]. As of May 2024, the XNAT platform has been decommissioned, and its services have migrated to the main OASIS Brains website. The OASIS Brains website allows users to browse, view, and access data directly. Users can then apply analytical platforms such as FreeSurfer for volumetric segmentation and structural analysis [141,142].

#### 3.1.3. The Alzheimer’s Disease Neuroimaging Initiative (ADNI)

The Alzheimer’s Disease Neuroimaging Initiative (ADNI) project [143] was launched in 2004 to develop and evaluate AD biomarkers. ADNI follows a public–private partnership model that brought stakeholders together to address complex health problems [144]. These stakeholders included Neil S. Buckholtz from the NIA and Dr. William Potter from Eli Lilly and Company. Their discussions helped lay the foundation for ADNI, which was funded by public agencies and private pharmaceutical partners to accelerate the development of effective AD treatments [145].

Building on our early discussion about the public–private partnership, NIH was also able to unite research expertise and funding, particularly through NIA, as well as the intellectual and financial support of leading pharmaceutical companies and non-profit organizations. The Foundation for the National Institutes of Health (FNIH) has also played a central role in coordinating this alliance by overseeing fundraising campaigns and acting as a neutral convener through the ADNI Private Partner Scientific Board (PPSB) [146]. Along with the PPSB, the NIH serves as an “honest broker” for balancing research interests between academic researchers and industry, which leads to maintaining integrity and transparency [147].

ADNI evolved through a series of phases. The first phase of ADNI was ADNI-1, which was launched in 2004. The initial cohort for ADNI-1 comprised individuals where 200 were cognitively healthy elderly adults, 400 participants were diagnosed with MCI, and 200 with early AD. The next phase of ADNI was ADNI-GO (2009–2011), which focused on shifting from its primary goal of analyzing biomarkers in the early stages of disease progression. ADNI-GO also implemented amyloid PET scans for continuing ADNI-1 participants and expanded the cohort by enrolling an additional 200 new participants classified with early MCI [148].

The next phase was ADNI-2 (2011–2016), whose primary objective of this phase was to further refine the utilities of biomarkers specifically focused on their development as robust ways to predict cognitive decline and assess outcomes from clinical trials. This expansion in ADNI was to capture the slightest of cognitive and biological changes to document clinical symptoms. ADNI-2 expanded its study population by 782 new participants, which includes a mix of elderly adult controls, early MCI, late MCI, and individuals with AD [149]. It was at this phase that ADNI became a powerful resource for researchers needing longitudinal data across different types of data for the development and validation of complex prediction models used for early disease prediction and studying disease progression [150].

ADNI-3’s main focus was on advancing neuroimaging techniques, including arterial spin labeling, diffusion MRI, tau PET, and task-free testing-state fMRI. This reflected the growing understanding of the role of tau pathology in neurodegeneration and its correlation with clinical and cognitive symptoms [151]. ADNI had a cohort of 1068 participants, including rollover participants from ADNI-2. The data were collected using 3T scanners [152]. More advanced imaging sequences introduced in ADNI-GO/2 were also integrated and updated into ADNI-3’s protocols. In ADNI-GO/2, resting-state fMRI was available mainly on Philips systems, whereas ADNI-3 task-free fMRI was more broadly available, with site-specific exceptions [153]. This phase in ADNI’s journey marked leadership and adaptability to state-of-the-art technological discoveries and improvements [154].

The current phase of ADNI’s lifecycle is ADNI-4, and it began enrolling participants for this cohort in 2023. It is expected to run until 2027. A major initiative undertaken by ADNI-4 is to address the historical lack of diversity in AD research cohorts [155]. The study aims to enroll around 1500 participants aged between 55 and 90. The 50–60% enrolled candidates are from populations that are historically underrepresented in AD research, including Latinx, Black, and individuals with less than 12 years of education, to address the demographic imbalance introduced in earlier studies, as well as recognized as a limitation that impacted the implementation of discoveries to a larger population [156].

To support accessibility, ADNI-4 introduced a “remote cohort”, which allows for online cognitive testing, blood-based biomarker collection, and other services. Remote cohorts marked a significant change in moving beyond traditional models of collecting all assessments through clinic visits [60]. ADNI-4 also created new tailored assessments that are designed to target social determinants of health, in order to record a more holistic view of how a person may develop a disease [157]. The standard MDI protocol includes structural sequences ASL, DTI, and task-free resting-state fMRI to allow researchers to combine perfusion, anatomical, and functional connectivity biomarkers [153,158]. This dataset also provides plasma biomarker data collection as well as digital neuropathology, providing digitalized slides of specific parts of the brain to approved users.

A strict inclusion and exclusion strategy was deployed for recruiting participants for the ADNI study. Participants were recruited between the ages of 55 and 90 years. As part of the data collection, there was a person called the “study partner” or caregiver, who is an individual responsible for providing an independent evaluation of the participant’s daily functions and must be available to accompany them to all scheduled study visits [159]. Key exclusion criteria are set in place to ensure that the observed changes are indeed a characteristic of AD pathology and not other conditions. These include the presence of metal implants incompatible with MRI [160], the presence of any major neurological disease other than AD, abnormal baseline MRI findings such as brain tumors or subdural hematomas, a history of major psychiatric disorders, recent history of substance abuse or alcohol, or any systemic illness that could interfere with the study.

For participants who were diagnosed with MCI or AD, additional protocols were applied, such as documenting memory compliance, keeping score on memory function [56], and specific score ranges in Clinical Dementia (CDR) scales and on MMSE. At the initial screening visit, participants are categorized into different diagnostic cohorts based on a thorough clinical diagnostic test. During the ADNI-GO and ADNI-2 phase, MCI cohorts were further divided into ‘early MCI’ and ‘late MCI’ to analyze the finer details in cognitive impairment [161]. However, these subcategories were later merged back into a single MCI classification during the ADNI-3 phase. The continuous refinement of biomarkers is essential for enhancing diagnostic accuracy and guiding therapeutic development.

The PET imaging data found on ADNI provide researchers with important molecular insights into AD pathology. The ADNI PET Core, a collaboration with the University of California, Berkeley, and the University of Michigan, is responsible for standardizing the quality control, preprocessing, acquisition, and analysis of all PET data [162]. The study initially began with Fluorodeoxyglucose (FDG-PET) imaging, which assesses the regional glucose metabolism as an indicator for neuronal function and dysfunction. ADNI also added amyloid tracers [163] like 18F-florbetaben (Neuraceq), 11C-PIB, and 18F-florbetapir (Amyvid) [164], to help with quantifying and detecting amyloid plaques, which accumulate decades before the onset of clinical symptoms. Tau tracers such as 18F-flortaucipir have been shown to be directly proportional to cognitive decline and neurodegeneration more than amyloid alone.

All scanners used in ADNI undergo a qualification process outlined in its PET Technical Manual [165]. Images uploaded to LONI then undergo rigorous pre-processing, which includes steps like averaging of frames to produce a single image, co-registration of dynamic frames for motion correction, standardization of image, voxel size, and uniform resolution achieved by smoothening images to a common resolution using scanner-specific 3D Gaussian filters. In 2023, all Aβ and tau ADNI PET data were re-processed by UC Berkeley using a 6 mm FWHM resolution to ensure consistency. After pre-processing, images were analyzed using FreeSurfer-segmented MRI scans co-registered to the PET data to provide regional measures in the Desikan–Killiany atlas [166].

ADNI also collects extensive cognitive, behavioral, and clinical assessments. With imaging (PET; structural, diffusion, perfusion, and resting-state functional MRI), fluid biomarkers (CSF, plasma), and genetic data, ADNI provides multimodal datasets to study AD progression. Core ADNI assessments include global screening and staging measures such as MMSE, MoCA, CDR/CDR-SB, and ADAS-Cog; memory tests such as Logical Memory and the Rey Auditory Verbal Learning Test (RAVLT); executive and processing-speed measures such as Trail Making; language measures such as Boston Naming and Category Fluency in earlier phases; and functional or behavioral instruments such as FAQ, ECog, NPI-Q, and GDS [56,167,168]. New tests were gradually introduced throughout different phases. For example, the American National Adult Reading Test (AMNART) was introduced in ADNI-3, the MoCA was introduced in ADNI-GO, and ADNI-4 added remote and digital screening, the Everyday Cognition 12-item measures, social-determinants-of-health assessments, and the Hollingshead Index [60]. These measures are important because ADNI models often use cognitive scores as outcomes, labels for diagnostic conversion, or non-imaging predictors combined with MRI, PET, CSF, plasma, or genetic biomarkers.

Genetic data also constitutes part of ADNI’s database. Fluid biomarkers were also an important data type that was part of ADNI. ADNI collected a wide range of biospecimens over time and changed its protocols over time to accommodate them [169]. The types of biofluids include plasma, serum, urine, and CSF. As CSF required patients to have a lumbar puncture, it was initially obtained from a subset of volunteers in ADNI-1. However, it changed over the later phases as it was obtained at screening for early MCI participants in ADNI-GO, and at baseline every 2 years for new MCI, AD, and control participants in ADNI-2 and ADNI-3. Compared to the obtained CSF, plasma, and serum samples were much easier to collect and were obtained from all participants across all ADNI phases with ADNI-4, even allowing for remote blood cohorts [170].

As far as database management and administration are concerned, ADNI was managed by LONI and IDA at the University of Southern California. The IDA securely de-identifies all ADNI data [171], and the data flow within ADNI is orchestrated by over 60 clinical acquisition sites and various analysis centers. Clinical data are collected using an electronic data capture system, while PET and MRI are uploaded from clinical sites within 24 h of acquisition. Then, the automation pipelines de-identify data, extract metadata from image headers, coordinate data workflows, and ensure cross-modality integration along with manual QC from analysts who ensure scan quality [172]. The de-identification processes ensure that ADNI datasets meet federal HIPAA standards for protecting participant privacy. To access data, users should use the LONI IDA website and request access, which is reviewed by the Data Sharing and Publications Committee (DPC) [173]. Once approved, the user must agree to the DUA. After all requirements are met, the data is released through the user portal in LONI IDA.

### 3.2. Genetics and Genomics Data

Genetics and Genomics play an important role in finding probable reasons behind AD and ADRD. While early-onset AD is often linked to inherited mutations like PSEN1 [174], PSEN2 [175], and APP [176], there are several other genetic as well as environmental factors that account for 70% of the risk a person might develop AD [177]. Recent advancements in [178] and sequencing have increased understanding by identifying various genetic loci that are associated with AD/ADRD [179]. Therefore, studies related to genetics have expanded our understanding of key biological pathways such as lipid metabolism, endocytosis, and inflammation [180]. Genetics and Genomics data hold high value in the scientific world as they could be used to develop precise medical approaches, enable identification of at-risk individuals, and improve diagnostic accuracy [181].

#### 3.2.1. UK Biobank

UK Biobank [182] was developed in the early 2000s, with formal participant recruitment occurring over four years (2006–2010) [183]. Its goal was to establish a comprehensive resource for studying the origins of common diseases and supporting prevention, diagnosis, and treatment research. Initial discussions began in 1999, followed by a preparation phase focused on ethical considerations, robust protocols, and funding. The investment stage included expert working groups focused on questionnaire design, physical measurement, sample handling, and recruitment methods [184].

The UK Biobank is open-access, meaning that the de-identified data is accessible globally to approved researchers from sectors like government, commercial companies, charitable organizations, and academia. The UK BioBank is widely known as one of the world’s most comprehensive large-scale health resources [183], containing data from almost 500,000 participants. The biobank is designed for long-term prospective study in order to “future-proof” studies related to disease progression over the decades, and to understand the long-term effects of lifestyle and environment.

During the recruitment, the participants for the study aged between 40 and 69 and were selected through sending an invitation to a pool of individuals registered under the National Health Service (NHS) across Wales, England, and Scotland [185]. Interested participants attended a 2–3 h assessment at one of the designated assessment centers. At these centers, patients gave consent to participate and then completed a questionnaire covering lifestyle factors, such as physical activity levels, smoking habits, alcohol consumption, and an 8-day dietary diary [186]. Next, a 1:1 interview between the participant and a nurse is conducted to gather more information on medical history, employment, number of children, marital status, and family history. A wide range of physical measurements, such as weight, blood pressure, height, grip strength, bone density, and lung function, were recorded along with a collection of biological samples such as blood, saliva, and urine using a Vacutainer system that was equipped with clot activators, preservatives, and anticoagulants [187].

Initially, processing at the assessment center was kept to a minimum to preserve quality. Once this is done, each vacutainer is inverted to mix its contents along with clot activator serum tubes and lithium–heparin plasmin to prevent degradation. Each sample is then barcoded and scanned to connect it with the patient ID. After this, samples are shipped overnight through a commercial courier to a centralized laboratory for further testing. At the central laboratory, multiple aliquots, such as red and white blood cells, whole blood, urine, and plasma, are prepared and stored at low temperatures. Next, hematology assays are performed on fresh blood samples while biochemical analyses are conducted on stored aliquots. By the end of the collection stage, 15 million aliquots are stored [187]. To ensure protection and long-term integrity, these samples are housed in two different archives.

The final participation rate after all these steps was 5.45% from the invited population group [185]. It is important to note that there is a self-selection bias that is common in volunteer-based cohort studies. This creates challenges when trying to evaluate the generalization of analysis to the broader UK population, creating what is known as the “healthy volunteer effect”. The “healthy volunteer effect” is a phenomenon that refers to the tendency of a research study to have participants who are more health-conscious than the general population. In the UK Biobank database, participants are more likely to be older, female, and to engage in healthier lifestyle choices. To combat this bias, UK BioBank developed Inverse Probability (IP) weights for the research community. These weights were estimated using the UK Census data to ensure they are representing the UK Biobank’s population. In action, IP weights substantially reduced volunteer bias, achieving an average reduction of 87% [188].

Participants who agree to data collection also agree to a long-term commitment to follow up with participants’ health extending for at least 30 years, during which information collected over time is integrated into their respective EHRs, as well as updating measurements over time [189]. UK Biobank maintains communications with its participants through dedicated helplines, official website, public meetings, and regular newsletters as part of not merely based on contract but also for building relationships with patients. Participants’ privacy is further reinforced by the database’s approach to consent, mostly through its “right to withdraw” policy, in which participants are given the options to choose from three levels: “No further contact”, “No further access”, and “No further access” [190]. UK Biobank is governed by UK GDPR rather than HIPAA, which ensures equivalent de-identification procedures, including secure linkage, participant-controlled withdrawal option, etc. With these options, UK Biobank supports the ethical integrity of prospective cohorts and ensures participant retention.

In 2014, the UK Biobank launched a project to perform detailed imaging on 100,000 participants, with plans for repeat scans in the future. The imaging dataset contains MRI scans of major organs, including the heart, abdomen, and brain, along with other imaging modalities such as carotid ultrasound for arterial health, optical coherence tomography (OCT) for detailed ocular data, and dual-energy X-ray absorptiometry (DXA) for whole-body measures of bone density and body composition [191]. For brain imaging, the UK Biobank includes T2-FLAIR, diffusion MRI, structural MRI, susceptibility-weighted imaging, arterial spin labeling (ASL), and both resting-state and task fMRI, enabling analyses of white-matter integrity, perfusion, and functional connectivity [192]. These data are available in several formats, including NIFTI, MAT, and DICOM files, and also include processed information known as image-derived phenotypes (IDPs), which are quantitative indicators generated through automated machine-learning pipelines. Examples of IDPs include left ventricular ejection fraction from cardiac MRI [193], visceral fat from abdominal MRI [194], detailed organ composition, and measures of white matter hyperintensities from brain MRI [195].

To process EHR data, the UK Biobank deploys data pipelines to connect participants to their EHR data from sources like the NHS [196]. These pipelines collect a wide range of clinical information, such as mortality and disease-registry feeds; inpatient records offering analysis into diagnoses, procedures, and hospital admissions; primary care records containing data related to prescriptions, referrals, and early-stage illnesses; and COVID-19 vaccination and test data that are useful for studying the impact and determinants of the virus [197]. To manage file format changes, handling incomplete or invalid records, and resolving linkage problems, the UK Biobank uses different tools and custom pipelines for individual feeds and actively keeps up with new data integration methods and architecture to streamline and standardize processing [189].

The genetic data available within the UK Biobank is one of its most powerful data resources. All 500,000 participants underwent genotyping, involving an analysis of almost 800,000 genome-wide variants, attributing to almost 90 million extra variants. This subset of data has been the source of many large-scale Genome-Wide Association Studies (GWAS), which are trying to expand our understanding of the genetic architecture of disease and complex traits [198]. Another layer of data is Whole Exome Sequencing (WES) [199], which was conducted for 470,000 participants. WES focuses on protein-coding regions called exomes, which are involved in protein production and are often a breeding ground for disease-causing mutations. The availability of WE = S data has been valuable for pharmaceutical companies in particular, since it helps them with identifying coding variants that are associated with a wide range of health-related traits. Finally, as part of the UK Biobank’s commitment to genetic data, it has also completed whole-genome sequencing (WGS) for its entire cohort [200]. To achieve this, an investment of over 350,000 h of sequencing [201].

Beyond biological samples, genetic data, and imaging, the UK Biobank also contains a variety of other data types. It has data on more than 30 key biochemistry markers, which are measured from blood samples that are collected at recruitment and the first recurring assessment for all patients [202]. The types of biomarkers include infectious disease markers, cholesterol, and data related to proteometabolic markers. A large-scale proteomics project is underway to measure protein levels in blood samples of all 500,000 participants, and this aims to build on a successful pilot study that has identified over 14,000 associations between protein expression levels and genetic variants. Another data type is the activity monitoring data that was collected for 100,000 participants, which was collected over a period of 8 days using wrist-worn accelerometers along with follow-ups [203]. UK Biobank also includes computerized cognitive testing collected through touchscreen and online tasks, including reaction time, pairs matching, prospective memory, numeric memory, fluid intelligence, trail-making, and related tasks, which can be linked with imaging, genetics, health records, and lifestyle variables for dementia-risk modeling [59,204]. Therefore, the UK Biobank is one of the best platforms for researchers to investigate AD/ADRD as it incorporates a large-scale integration of brain imaging, genetics, and many other forms of data. The cohort’s diversity and size, along with the implementation of Inverse Probability to help adjust sampling bias [205], have enhanced the ability to generalize findings to the broader population.

#### 3.2.2. Database of Genotypes and Phenotypes (dbGap)

The database of Genotypes and Phenotypes (dbGaP) [206] is a central NIH repository for disseminating and archiving studies of phenotype-genotype relationships. It includes GWAS, molecular diagnostic assays, medical sequencing, and research on relationships between non-clinical traits and genetic makeup. dbGaP was founded in 2006 in response to NIH’s GWAS policy [207]. Its creation addressed rapid advances in cost-effective genotyping [208] and sequencing, which generated unprecedented volumes of genotypic data. The database also supported NIH’s policy of treating dbGaP data as a pre-competitive resource, maximizing public scientific value while discouraging restrictive intellectual-property claims.

To submit, the data collection process starts with identifying a subject appropriate Genomic Program Administrator (GPA) that falls under the relevant funding center or institute [207]. Individual investigators are not permitted to register data on their own. One of the cornerstones of the submission process is regulatory compliance, so researchers are required to obtain an Institutional Review Board (IRB) review along with a signed Institutional Certification [209]. This certification serves as an assurance to NIH that the data submission adheres to the Genomic Data Sharing (GDS) policy, institutional policies, and informed consent provided by participants in the study.

During the data preparation stage, data submissions must provide individual-level genotyping and phenotyping information. These data often include multimodal elements such as genetic variants, environmental exposures, and clinical traits, depending on the cohort. This data is sensitive and is then de-identified using a random unique code with a key that should be controlled by the submitting institution [209] to maintain participant anonymity. dbGaP enforced HIPAA guidelines for de-identification and operates under the NIH’s Genomic Data Sharing (GDS) policy. Along with the main data, researchers should submit metadata along with study protocols, questionnaires, manuals of procedures, and a detailed data dictionary that defines measured variables along with their measurement methodologies [207]. On the outer scale of things, the GPA initiates study registration with dbGaP, and the Principal Investigator (PI) of the study completes a detailed study information form, followed by the GPA’s verification. Finally, if all protocols are approved, the PI submits the data to dbGaP.

It is important to note that the management and control of data that is hosted in dbGaP still remains under the jurisdiction of the sponsoring institute and the original study. One significant drawback of the dbGaP database is that the multi-step process is heavily institutionally dependent, along with problems related to user feedback, such as “lack of proper instruction” and need for “multiple revision”, which could be an administrative burden leading to inconsistencies and delays in data entry stages, impacting ease of use and standardization for external researchers [210].

dbGaP uses a two-tiered system to balance data utility with privacy protection [211]. The first tier is open access, where general metadata such as descriptions of measured variables, study summaries, and original study documents are publicly available. The second tier is controlled access, which includes sensitive individual data such as genotypes, phenotypes, and pre-computed univariate associations. Access to controlled data is granted by the NIH Data Access Committee (DAC). Researchers seeking access submit a Data Use Certification (DUC) through an NIH eRA Commons ID [212]. The DUC ensures that the proposed research aligns with the participant consent terms under which the data were collected. Once approved, users receive controlled access governed by strict terms and conditions.

#### 3.2.3. The National Institute on Aging Genetics of Alzheimer’s Disease Storage Site (NI-AGADS)

NIAGADS, the National Institute on Aging Genetics of Alzheimer’s Disease Data Storage Site, was established in 2012 through an agreement between NIA and the University of Pennsylvania [213,214]. Its mission is to provide qualified investigators with access to late-onset AD genetics data. NIAGADS addresses emerging challenges in AD genetics research by integrating richer phenotypic datasets, increasing sample sizes, and supporting population-specific disease-risk models. NIAGADS is not another name for ADSP. Rather, NIAGADS is the designated national data repository and Data Coordinating Center (DCC) that supports AD/ADRD genetics data. ADSP, by contrast, is the Alzheimer’s Disease Sequencing Project, a major NIH-launched sequencing initiative designed to identify genetic risk and protective variants for Alzheimer’s disease [215]. Because the two are tightly linked in AD research, their identities may appear to overlap: NIAGADS supports ADSP data production, management, and distribution, while ADSP generates and harmonizes many sequencing datasets deposited through NIAGADS.

NIAGADS data-sharing policies comply with the Federal Information Security Management Act of 2002 (FISMA) and HIPAA [216]. The NIAGADS Data Sharing Service (DSS) operates within a FISMA Moderate-compliant cloud platform, and the stored data are controlled and governed by the NIH Ge-nomic Data Sharing (GDS) policy [217]. NIAGADS includes genomic, genetic, and phenotypic data enhanced by integrated neuropathology and clinical data. The integration of multiple data types makes NIAGADS a valuable multimodal resource for investigating phenotypic and genetic correlations in AD. The DSS houses 20,503 whole exomes and 58,507 whole genomes from ADSP and other ADRD studies. All genomic data in NIAGADS are mapped to the GRCh38 human reference build to ensure consistency across datasets [218]. NIAGADS also provides summary statistics from published genetic studies through its Open Access platform. Cognitive and behavioral phenotypes in NIAGADS generally come from contributing cohorts and ADSP phenotype harmonization rather than direct testing by NIAGADS itself; common variables may include diagnosis, age at onset, cognitive status, and cohort-specific scores such as MMSE or CDR when available.

NIAGADS currently serves as the secure portal through which researchers access, download, and analyze ADSP genomic datasets and other NIA-funded AD/ADRD genetic studies. Access to sensitive datasets such as ADSP WGS and individual GWAS is controlled [219]. To access data, PIs initiate a formal Data Access Request (DAR) through the Data Access Request Management (DARM) system using their eRA Commons ID for authentication. PIs may designate an administrator to submit a DAR on their behalf, provided that the administrator has an eRA Commons ID. A DAR requires a project description and contact information for the institutional signing official, PI, and IT director. The user must also submit a Research Use Statement (RUS), a concise non-technical summary for public circulation, a Secondary/Derived Data Return Plan, IRB approval and rating, an NIA Genomic Data Sharing Plan, and the NIAGADS Data Distribution Agreement [220].

Once all these documents are submitted, the application undergoes review under the NIAGADS ADRD Data Access Committee (NADAC) and the Data Use Committee (DUC). To ensure responsible use of data, applicants must be permanent employees at their institution and hold a position equivalent to a full-time assistant or associate [221]. All data within DSS are access-controlled and governed by NIH Genomic Data Sharing (GDS) policy. Approved users should formally agree to follow the NIH Genomic Data User Code of Conduct. This agreement requires that investigators use the NIAGADS datasets exclusively for the research project described in the approved DAR. Downloaders of the data should also sign a Data Transfer Agreement (DTA) and review the Data Use Certification Agreement.

The DSS is housed on Amazon Web Services (AWS), and researchers can access data through the Data Portal, download it directly, or work with it through AWS Elastic Compute Cloud (EC2) [222]. Under NIA policy, data derived from NIA-funded studies related to late-onset AD genetics should be stored in NIAGADS or another NIA-approved site [223]. The process starts by emailing required documents to help@niagads.org, including an Institutional Certification for ADRD studies, a signed NIA AD Genomics Sharing Plan, and a Data Registration template.

For each individual data entry, the following information is needed: platform used, concise description of the study design, relevant version information, corresponding data dictionary, complete list of included formats and files, contributor contact information, the dataset’s reference genome build, and any associated publications. All data submissions are required to have MD checksums, a 128-bit digital fingerprint for data files that is generated by a cryptographic hash function to produce a unique hexadecimal string from any input to ensure that the data is free from incomplete transfer, unintended alterations, and corruption, and a README file in PDF, Microsoft Word, or plain text format.

Once data are submitted, all ADSP data entering NIAGADS are first routed through NIAGADS to validate data-transfer agreements and ensure compliance with the original informed consent for data flow and sharing activities. Depending on the data received, the data are forwarded to a consortium for further processing. NIAGADS collaborates with 73 ADSP cohorts spanning 31 countries across 6 continents. As part of data submission, cohorts can either submit DNA samples directly to NCARD for quality control and sequencing preparation or provide completed WGS data as raw sequence reads [224].

Beyond collaboration with cohorts, NIAGADS works with other NIA infrastructure, such as NACC and the AD Knowledge Portal, for data integration [225]. NIAGADS is a data coordinating center, meaning it orchestrates data flows among partners. These partners include TAGC/HIHG for sequencing, GCAD for variant calling, NCARD for biological samples, and ADSP-PHC for phenotype harmonization. These data flows help NIAGADS maintain consistency, minimize system errors, and provide AD/ADRD research data.

### 3.3. Drug Discovery and Therapeutics Data

Drug Discovery and Therapeutics play an important role in the fight against AD and ADRD. It lays the foundation for scientists to identify potential interventions, slow down disease progression, and improve patient outcomes. AD is characterized by its wide spectrum of neurodegenerative conditions, which lack treatment. Drug Discovery and Therapeutics focuses on finding databases that contain data related to molecular targets such as tau proteins, amyloid proteins, and synaptic dysfunction, so that by leveraging these datasets, researchers can design monoclonal antibodies, design small molecules, or create gene therapies that can interact with these biomarkers [226]. The integration of therapeutics development with preclinical models allows researchers to make simulations of disease progression and test the efficacy across biological systems mimicking human neuropathology [227]. Since AD/ADRD are difficult to diagnose, precision medicine is currently being explored to tailor medicines to an individual’s genetic, environmental, and lifestyle profiles.

#### 3.3.1. Chemical Entities of Biological and Medicinal Interest (chEMBL)

The Chemical Entities of Biological and Medical Interest (ChEMBL) database [228] is a bioactive molecule database developed by the European Bioinformatics Institute. Earlier, this database was known as StARlite, and it addressed the need for structured bioactivity data in drug discovery and therapeutics. In 2008, ChEMBL was acquired by the European Molecular Biology Laboratory (EMBL) through an award from the Wellcome Trust [229]. This acquisition helped democratize access and lowered barriers for researchers across industry and academia. ChEMBL version 2 was launched in 2010 with 2.4 million bioassay measurements, 24,000 natural products, and 622,824 compounds curated from over 34,000 publications. ChEMBL bridges biological activity, genomic information, and chemical space by storing bioactivity data against drug targets with measurements reported as Kd, IC50, EC50, and Ki [230]. These bioactivity profiles are linked to protein targets, chemical structures, and assay metadata, making ChEMBL an important multimodal dataset for drug discovery. ChEMBL does not contain personally identifiable health data, so HIPAA does not directly apply. The data can be analyzed and filtered into focused compound-screening libraries for lead identification, drug-selectivity analysis, off-target assessment, and predictive modeling [231].

ChEMBL is an important component of the ELIXIR infrastructure [232], which is an intergovernmental organization that unites Europe’s life science organizations to provide sustainable services and data resources. ELIXIR ensures that ChEMBL gets sustained funding, seamless integration with other biological and chemical initiatives, and fosters a collaborative environment. AN independent impact report from a survey sent to over 4000 users, ChEMBL was ranked 5th among EMBL-EBI’s most used resources.

A key strength of ChEMBL comes from its structured data model, which centers around entities such as chemical compounds, biological targets, experimental assays, and quantitative activity data [233]. Compounds within ChEMBL are collected for their drug properties and categorized into approximately 14,000 clinical candidates, 4000 approved drugs, and 2.4 million preclinical compounds. For each of the compounds, ChEMBL offers 2D chemical structures and a suite of physicochemical properties like molecular weight, Lipinski parameters, and LogP, which are crucial for predicting psychokinetic behavior and assessing drug-likeness [234]. As of recent, Release 32 has also expanded coverage for newer therapeutic classes such as oligonucleotides, gene therapies, and antibodies.

For the target, ChEMBL includes 17,000 unique biological targets, and most of the protein-based targets [235] are categorized into ‘single protein’, ‘protein complex’, etc. It also accounts for non-protein targets as well, such as metabolites, nucleic acids, and whole organisms. For assays, ChEMBL organizes them into six categories: functional, binding, toxicity, ADME, unclassified, and physicochemical, each of which has detailed metadata that provides context about the experiment, the target identifiers, and the organism.

ChEMBL is architecturally built on a relational database system, which allows for efficient retrieval, complex querying of vast biological and chemical data, and systematic storage [236]. At its core, ChEMBL’s data is the experimentally determined association between a ligand (chemical compounds) and the biological target [237]. The database uses a series of key tables to connect different entities within the ChEMBL. Information related to chemical compounds is housed in the ‘molecule dictionary table’, which is linked to ‘molecule properties’ for physicochemical attributes and ‘molecule form’ to manage the relationship between salt forms and parent compounds. Quantitative bioactivity measurements are stored in the activities table, while the assay table provides detailed information on experimental protocols, and biological targets can be found within the ‘target dictionary’ [233].

An important feature of ChEMBL’s schema is its granular annotation of binding sites [238]. These are defined in relation to a target that can be specified at multiple levels of detail, such as a specific domain within that protein, precise amino acid residues that constitute the binding pocket, or even a specific domain within that protein. ChEMBL routinely conducts internal research projects to identify possible binding domains of each target. For a subset of activities, predicted compound-binding Pfam domains [230] are annotated and integrated into the data model to provide structural context for the bioactivity data. To facilitate the usage of computational developers and scientists and to ensure transparency, the ChEMBL release schema diagram is made public and can be directly accessed through ChEMBL to offer a dynamic, exorable representation of the database structure.

ChEMBL’s data quality depends on a rigorous and systematic curation process. This includes careful identification, evaluation, and organization of information extracted mainly from experimental results and peer-reviewed scientific literature [231]. The curation process begins with identifying scientific facts within journal articles, after which the extracted data undergo formatting and standardization before integration into the database. For publications that include Structure-Activity Relationship (SAR) data, such as IC50 values for compounds targeting specific proteins, the ChEMBL selection process takes steps to ensure consistency and precision. First, it translates graphical representations, scaffolds, or R-group tables from the original articles into machine-readable chemical structures using formats such as Simplified Molecular-Input Line-Entry System (SMILES) strings or molfiles [239]. The system then identifies the appropriate UniProt accession number [240] for the protein target by specifying the source organism and extracts a descriptive summary of the experimental assays in which bioactivity was observed and measured. This structured dataset is then added to ChEMBL to ensure that chemical structures are consistently annotated and biologically contextualized.

The ChEMBL web interface serves as the primary portal to the database’s bioactivity data through encrypted and secure protocols [241]. One of its key features is its flexible text matching feature, which allows users to perform powerful free-text searches across all major subjects in ChEMBL, including targets, compounds, assays, cells, documents, and tissues [242]. An autocomplete feature is also available to give real-time suggestions and guide them to what they are searching for. Auto complete is supported by the Elasticsearch engine and supports a custom domain-specific language (DSL) to enable users to create highly complex queries for retrieving specific information. For chemists, an integrated Marvin JS chemical sketcher is available for users to draw chemical structures directly on the interface to help with similarity and substructure searches. The results can then be visually enhanced through structure highlighting, making it easier to explore chemical space based on structural motifs. Researchers can also search by protein sequence similarity using the EBI-wide BLAST tool [243]. BLAST is especially useful for identifying the related proteins that are associated with activity data. ChEMBL identifiers (IDs) allow users to easily retrieve multiple known subjects at once, allowing for batch analyses.

Beyond search, ChEMBL also supports data visualization. ChEMBL features an interactive “circles” visualization on its main page summarizing ChEMBL quantities and entities. Users can click on these ‘circles’ to explore different entities to get a hint of what the database offers. Browse pages and search results are equipped with a filter on the left side to visually represent data distribution and help users refine their results by different properties [234]. For each target, compound, assay, and document, comprehensive ‘report cards’ are also available, which provide information, such as chemical structures, synonyms, cross-references, and calculated physicochemical properties and references to other external resources. ChEMBL also uses descriptive URLs that accurately reflect the data being displayed, easy sharing of search results, and reproducibility of queries among researchers.

On the computational side, ChEMBL offers programmatic access through RESTful web services, allowing for remote retrieval of ChEMBL data and integrating it into custom applications and workflows [234]. The API can provide access to a range of ChEMBL entities, and each entity has its own endpoint. Data exported can be of different formats, such as JSON (default), XML, and TAML. Chemical structures can be exported as MDL molfile format, and images of molecules are available as SVG or PNG. The API also supports HTTP POST and GET methods. For reference, a Python client library called ‘chembl websource client’ is also available, and it offers documented use cases for all functionalities, removing any complexities researchers may have with API calls [244].

For users who need high-throughput querying without the fear of rate limits or integration of proprietary data, ChEMBL provides an option to download data in bulk using file transfer protocol (FTP). Bulk download options come in several formats that are tailored to different technical needs [245]. Entire relational database dumps of ChEMBL can be accessed through MySQL, PostgreSQL, and SQLite formats. These formats include data from ChEMBL but require users to manage a local database instance, making them ideal for executing complex queries across multiple tables. For a more lightweight access, ChEMBL provides flat files such as Structure Data Files (SDF) for compound structures, FASTA files for protein sequences, and versioned release notes highlighting changes and updates. A Resource Description Framework (RDF) of the database formatted in Turtle is available to enable linked-data querying and seamless integration with other RDF-enabled datasets [245].

Beyond the database, the ChEMBL group at EMBL-EBI has developed a suite of resources and tools. Kinase SARfari is an integrated chemogenomics tool for the protein kinase family of drug targets. It consolidates and links protein structures from PDB, chemical compounds, kinase sequences, and screening data. GPCR SARfari is another ChEMBL resource focused on G-protein-coupled receptors (GPCRs), another major class of drug targets [246]. ADME SARfari is a ChEMBL web resource that supports comparative analyses of drug-disposition genes [247]. It integrates public data sources for data mining, visualization, and predictive tools for drug metabolism. A key feature of ADME SARfari is its in silico models for predicting which ADME-relevant protein targets a novel compound may interact with. myChEMBL is a free virtual machine (VM) from ChEMBL that combines the PostgreSQL version of the database with the chemistry cartridge and RDKit cheminformatics toolkit [248]. The VM offers local access to ChEMBL Web Services, a chemical-search web interface, and interactive IPython notebook tutorials. SureChEMBL is a public resource that extracts compounds from patent documents using an automated pipeline and supports combined structure- and keyword-based searching [249].

#### 3.3.2. DrugBank

DrugBank Online [250] is an open-science database designed to integrate information about drugs and their biological targets [251]. It is widely used by pharmacists, physicians, scholars, and the public. Unlike many chemical databases that focus mainly on compound structures, DrugBank links chemical, pharmaceutical, target, sequence, pathway, and pharmacological information. Clinically oriented sources such as RxList and PharmGKB contain rich clinical details, including metabolism, indications, and pharmacology, but they are often limited in chemical, structural, or physicochemical data. Drug-Bank addresses this gap by supporting both bioinformatics and cheminformatics analyses and by linking drug targets to structural characteristics, sequences, and biological pathways [252].

The DrugBank database began in Dr. David Wishart’s laboratory at the University of Alberta in 2006. Its objective was to help researchers access structured and organized drug information. In 2011, the project gained support from The Metabolomics Innovation Centre (TMIC), a nationally funded research facility. The database evolved in response to user requests and changes in drug research and development [253]. Early versions of DrugBank added fields for absorption, distribution, metabolism, excretion, and food-drug interactions. Later versions, such as DrugBank 4.0, added Quantitative Structure-Activity Relationship (QSAR) data to support predictive modeling in drug design [254].

As of January 2025, DrugBank holds about 17,476 total drug entries, which include 1745 approved biologics data (proteins, vaccines, peptides, and allergenics), 6878 experimental (discovery-phase) drugs, 135 nutraceuticals, 3000 approved small molecule drugs, and 5467 non-redundant proteins. More than 200 data fields. Older versions of DrugBank also included drugs that are illicit or have been withdrawn from markets [251]. Each entry in the database is called a “DrugCard,” and it contains more than 200 data fields, of which half of the information is related to drug/chemical data, while the other is for protein data or drug data. These fields span chemical, genomic, and clinical data, making the database a multimodal platform for therapeutic research.

The two main types of information available in DrugBank are drug target/receptor information and drug/compound information [255]. Drug target/receptor records provide a target’s protein sequence, number of residues, Gene Ontology (GO) annotations, molecular weight, biochemical reactions, and pathways. More specific molecular details are also available, such as signal sequences, transmembrane regions, Pfam domains, and target-essentiality assessments. These fields include QSAR data that can support predictive computational models.

DrugCards also contain drug/compound information covering pharmacological and chemical attributes. These include IUPAC nomenclature, molecular weight, chemical formulas, pKa or pI values, LogP or hydrophobicity measurements, melting point, detailed chemical sequences and structures, water solubility, and brand names. DrugCards also include Mass Spectrometry (MS) spectra [256], Nuclear Magnetic Resonance (NMR) data, SMILES strings, and file formats such as SDF, MOL, and PDB for computational and structural analysis.

DrugBank 5.0 marked significant progress by adding qualitative information on how drugs influence protein, gene expression, and metabolite levels. For a given drug, DrugBank can provide drug-protein, drug-transcript, and drug-metabolite interactions. Each data field indicates whether the drug decreases or increases expression for the respective biological entity and links the assertion to a supporting literature reference [252].

DrugBank Online operates on a hybrid access model [257]. Resources are generally accessible through the online platform, but redistribution or use of DrugBank content for a specific purpose requires an appropriate license. Academic users may apply for a free license for non-commercial research and educational use, while commercial entities or users requiring application-specific data must obtain a paid license [258]. DrugBank data are gathered from publicly available sources, and each data point can be traced to its original source. As of January 2025, DrugBank reported about 17,476 total drug entries, including 1745 approved biologics, 6878 experimental drugs, 135 nutraceuticals, 3000 approved small-molecule drugs, and 5467 non-redundant proteins. Older versions of DrugBank also included illicit or withdrawn drugs.

DrugBank data curation is performed by experts in bioinformatics, pharmacy, and cheminformatics. Validation and compilation involve systematic searches across journal articles, textbooks, and electronic databases. Each database entry is called a DrugCard and contains more than 200 data fields; about half relate to drug or chemical data, and the remainder relate to protein or target data [259]. Each DrugCard undergoes two-step validation: one curation-team member prepares the DrugCard, and a second independently validates it. Senior members of the curation group, including PhD-level biochemists and experienced physicians, routinely spot-check entries. DrugBank has also integrated AI and software tools to improve precision and efficiency during curation [260]. Specialized packages, such as chemical-parameter calculators, text-mining tools, and protein-annotation tools, assist curators. DrugBank uses in-house experts to verify and review AI-generated outputs.

When it comes to data sourcing, DrugBank runs a screen-scraping tool on authoritative databases [261] and bodies like the FDA, Protein Data Bank (PDB), and RxList websites. Older and more obscure drugs are collected through manual curation efforts. Once the drug targets and all drug structures are identified and confirmed, they undergo cross-referencing with multiple sources like PubMed, RxList, etc. DrugBank also maintains an updated schedule for updating its information. DrugBank provides “DrugBank downloads” datasets, which are released quarterly for commercial and academic licenses.

DrugBank Online offers a freely accessible web interface that serves as the main portal for accessing data. For text-based searches, DrugBank uses a powerful search engine based on the Lucene query language. Lucene enables Boolean logic, wildcard searches, parenthetical groupings, exact phrase matching using quotation marks, and field-specific searches. Beyond text searches, the interface provides browsing options through PharmaBrowse and Browse. For specialized biological inquiries, DrugBank offers Local BLAST search (SeqSearch) [261], enabling users to identify potential drug leads by searching against DrugBank’s database of known drug-target sequences. A data extractor tool is also available for constructing complex SQL queries through user-friendly web forms. Recent versions include New Smart Search, a feature that supports filtering, ordering, and in-line previews. The Advanced Table Builder allows users to develop and save custom data tables to answer complex questions across datasets.

DrugBank also developed a Clinical API to facilitate integration with software applications and healthcare systems [256]. The API follows REST principles, uses standard HTTP response codes for errors, implements predictable URLs, and returns data in JSON format. DrugBank API access is managed through API keys that offer unlimited call capacity, while development keys are capped at 3000 requests per month. The API also supports browser-based applications. All API requests should be made over HTTPS to ensure secure data transfer [252]. The API supports regional filtering, meaning queries can be filtered by geographic region so that returned product information is relevant to a particular market and regulatory context. Pagination is enabled by default to optimize response times [262]. Users can control the number of results per page and navigate through pages using the page parameter. The API also offers a wide range of endpoints for accessing drug information.

When it comes to exporting data, DrugBank has multiple options for downloading datasets. Through DrugBank Open Data, users can access data that is free. It includes DrugBank structures containing names, structures, and synonyms for easy linking and integration, along with DrugBank Vocabulary containing names, synonyms, and identifiers. DrugBank supports most standardized formats such as SDF, CSV, and XML [251].

Therefore, the eight databases reviewed—clinical and population cohorts (NACC, OASIS, ADNI), large-scale genomics and genetics resources (UK Biobank, dbGaP, NIAGADS), and drug discovery or therapeutic repositories (ChEMBL, DrugBank)—build a foundational backbone for modern AD/ADRD research. NACC and ADNI emphasize longitudinal multimodal data, OASIS supports open imaging, UK Biobank provides population-scale genetic and imaging depth, dbGaP and NIAGADS enable reproducible genomic discoveries, and ChEMBL and DrugBank accelerate translational therapeutic research. At the same time, these databases face challenges, including demographic imbalances, metadata inconsistencies, controlled-access bottlenecks, and limited integration across modalities. Table 1 summarizes representative scale or impact indicators for the eight reviewed AD/ADRD databases, and Table 2 summarizes data formats used across these databases.

## 4. Potential Real-World Applications

Across the eight databases, impact and popularity vary by resource type. ADNI and NACC are heavily used clinical and multimodal cohorts; OASIS supports open neuroimaging research; UK Biobank, dbGaP, and NIAGADS support population-scale genetics and genomics; and ChEMBL and DrugBank support drug-discovery analyses. Because publication totals and citation counts change over time and are not reported uniformly across resources, this review reports representative scale or impact indicators rather than ranking the databases by a single publication-count metric.

### 4.1. Early Diagnosis and Risk Grouping Using Multimodal Data

Accurate and early diagnosis of AD/ADRD is critical for timely intervention [30]. Because these diseases are complex, diagnosis benefits from multimodal integration of cognitive assessments, fluid biomarkers, neuroimaging data, and clinical histories [41,263]. Databases such as OASIS, ADNI, and NACC provide longitudinal, standardized, multimodal data that can be used to validate and develop robust stratification and diagnostic models [33,106,126]. NACC provides clinical, pathological, and imaging data, allowing researchers to associate symptoms with post-mortem and imaging findings [107]. OASIS provides imaging modalities such as DTI, FLAIR, SWI, PET, and T1w imaging, along with clinical and cognitive data for studying connectivity, brain structure, and pathology [132]. OASIS-4 extends this resource to clinically evaluated individuals with early symptoms by combining CSF biomarkers, neuroimaging, and neuropsychometric evaluations [124]. ADNI focuses on biomarker validation through standardized cognitive tests, fluid samples, and rigorous imaging collection [144,169]. Together, these databases provide a strong foundation for research into AD/ADRD progression and early detection through standardized, multimodal data and AI-driven diagnostics [81,139].

In the field of MCI research, a majority of research conducted gives importance to subjective cognitive decline (SCD). SCD is where individuals self-report their worsening of cognitive function despite their scoring ranges being normal within standardized tests. SCD is recognized by scholars as a potential preclinical stage of AD, as longitudinal studies have shown SCD can precede measurable impairment and precede biomarkers. To address this, several large-scale initiatives have been set in place. The Subjective Cognitive Decline Initiative (SCD-I) Working Group has established protocols and harmonized datasets to enable cross-cohort comparisons in AI/ML developments [264]. A recent study has shown evidence that SCD populations tend to show a higher risk of progression to dementia and MCI, highlighting their value in early diagnosis frameworks. Therefore, when considering a multimodal approach, researchers could look into integrating SCD-related data into other data to significantly improve risk stratification and early diagnosis.

### 4.2. Development and Validation of AI/ML Models for Clinical Decision Support

**Figure 2:**
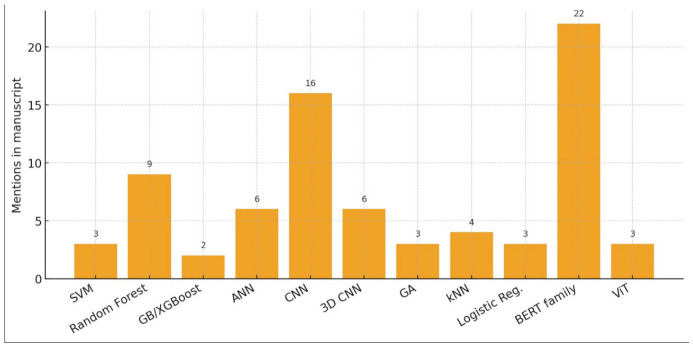
Algorithms discussed in AD/ADRD research papers included in this review (2000–2025). Values are reported as algorithm-mention counts from the review corpus.

Due to the multimodal nature of population and clinical health data, manual analysis is difficult and time-consuming [82]. Computational tools such as ML and AI can extract hidden patterns, support automated clinical decisions, and help predict disease trajectories [16,263]. Databases such as ChEMBL, UK Biobank, and dbGaP provide diverse datasets that serve as training grounds for robust models [183,207,229]. Techniques such as SVM, ANN, RF, AdaBoost, GA, CNN, ViT, ClinicalBERT, Blue-BERT, and KNN are widely applied in studies using resources such as the Mayo Clinic Study of Aging, AIBL, and ADNI [20,31,32,33,265]. Figure 2 summarizes algorithm mentions in the studies reviewed here. GA-based methods help navigate large search spaces to identify informative features for disease diagnosis and progression modeling [266], while modern variants incorporate multi-objective optimization, elitism strategies, adaptive crossover and mutation rates, and hybrid models that combine GA with deep learning or local search. In medical imaging, ViT and CNN models have demonstrated success in early detection using image data [38,78]. For unstructured data, NLP models such as BlueBERT and Clinical-BERT can capture physician observations that are not represented in structured data [69,70]. Novel 3D CNN architectures have also been developed to integrate structured and unstructured data [136]. These innovations can increase data utility and support AI-driven decision support and early detection [81,139].

### 4.3. Personalized Medicine

As our understanding of genetic heterogeneity changes, the potential for personalized medicine has increased [181]. By studying relationships between genetic profiles and therapeutic outcomes, large-scale studies can help identify adverse reactions, estimate drug efficacy, and guide individualized treatment development [179,180]. Genomic and genetic data in these datasets not only refine diagnosis but also support precision medicine [217]. For example, UK Biobank includes whole-exome sequencing (WES), which is valuable for identifying coding variants associated with health traits [199]. Because proteins are often involved in drug metabolism, proteomic analysis is foundational for pharmacogenomic applications [247]. dbGaP further supports this work by linking phenotypes and genotypes, enabling researchers to map genetic variants to drug responses or adverse events [207,212]. Integrating genomic, proteomic, and phenotypic data across databases can create a powerful framework for predicting how individual patients may metabolize or respond to AD therapies [216,226].

### 4.4. Understanding Drug Mechanisms of Action and Off-Target Effects

Understanding drug mechanisms and potential off-target effects is important for minimizing adverse reactions, optimizing efficacy, and designing safer therapies [267]. Databases such as ChEMBL and DrugBank provide detailed annotations for compound binding sites, amino acid residues, and Pfam domains, helping researchers identify where and how a compound interacts with its target [231,252]. This information is important for explaining a drug’s mechanism of action and supporting rational drug design [233,254]. These databases also support efficacy testing and preclinical model development. DrugBank information on pathways, biochemical reactions, target essentiality, and drug-target relationships can help researchers choose in vivo and in vitro models that accurately represent AD-related neuropathology.

## 5. Conclusions

ADRD represents a significant global health challenge, and its diagnosis calls for advancing research tools and collaborative efforts to improve treatment, diagnosis, and understanding of these conditions. By categorizing different databases based on their usage in research, researchers can identify potential datasets that could be tailored to their specific needs. Today, researchers use multiple computational methodologies to change how we analyze AD/ADRD data. Techniques like NLP, ML, and biomedical imaging have enabled researchers to extract meaningful patterns from complex datasets, which could be used to facilitate the development of predictive models, risk stratification, and early diagnosis. The success of algorithms like 3D CNNs, GA, and ClinicalBERT has shown the potential of using artificial intelligence to process heterogeneity and high-dimensionality of AD/ADRD data.

Databases such as ADNI, UK Biobank, and NIAGADS offer powerful, large-scale, longitudinal datasets that support AD/ADRD research. These repositories provide rich datasets while also addressing historical gaps in representation, including the inclusion of underrepresented populations. Their ethical frameworks and standardized pipelines help ensure data accessibility, quality, and privacy, allowing researchers worldwide to contribute to and benefit from open science. These databases also support personalized medicine and improved clinical outcomes. Using genetic, phenotypic, and proteomic data, researchers can predict drug responses, identify biomarkers, and tailor therapies to individual patients. DrugBank and ChEMBL further support drug discovery by clarifying mechanisms of action and off-target effects, helping advance novel therapeutics.

In the future, datasets should move beyond data collection alone and embrace designs optimized for AI-driven analysis. This can be achieved by including underrepresented populations to address equity in AD research, embedding standardized metadata, and ensuring longitudinal follow-up. Recent biotechnology advances, such as ultrasensitive biomarker assays [268], wearable sensor technologies [269], and CRISPR-based functional genomics [270], will enable datasets to reflect both real-world and biological variation. By aligning database architecture with modern AI and biotechnology needs, the research community can accelerate progress toward personalized therapies and early diagnosis.

In conclusion, AD/ADRD databases are valuable resources in the fight against neurodegenerative diseases. By centralizing and standardizing data and promoting open science, these resources empower researchers to study complex AD/ADRD mechanisms with precision. As neurodegenerative disease research continues to evolve, investment in computational tools, data infrastructure, and collaboration will be essential for developing effective interventions for AD and related dementias. This paper helps researchers identify datasets and computational methods for AD/ADRD studies, especially data-science projects involving AI.

## Figures and Tables

**Table 1:** Representative scale or impact indicators for the eight AD/ADRD databases reviewed.

Database	Representative Scale or Impact Indicator
NACC	Longitudinal UDS, neuropathology, imaging, and genetics data from NIA ADRCs
OASIS	Open MRI/PET and clinical/cognitive datasets for normal aging and AD/ADRD research
ADNI	Large multimodal AD cohort with imaging, fluid biomarkers, genetics, and cognitive assessments
UK Biobank	Population-scale health, imaging, genotyping, exome, and phenotype resource
dbGaP	NIH genotype–phenotype archive for controlled-access genomic and phenotypic study data
NIAGADS	AD/ADRD genetics repository and ADSP access point, including WGS/WES and harmonized phenotype data
ChEMBL	Curated bioactivity, compound, assay, and target data for drug-discovery modeling
DrugBank	Drug–target knowledgebase integrating drug, protein, pharmacology, and interaction data

**Table 2:** Data formats used across the eight AD/ADRD databases.

Database	Domain	Data Formats/Standards
NACC [104]	Clinical, pathology	Uniform Data Set (UDS); structured forms (CSV/Excel); not fully standardized across sites
OASIS [124]	Imaging	BIDS standard; MRI/PET data in NIFTI; metadata in JSON
ADNI [143]	Multimodal (imaging, fluid, genetics)	Imaging in DICOM/NIFTI; fluid assays in CSV; genetics in PLINK/VCF; cognitive data in tabular files
UK Biobank [182]	Population genetics and imaging	Imaging in NIFTI; genomics in VCF/PLINK; phenotypic and lifestyle data in CSV/TSV
dbGaP [206]	Genotype–phenotype archive	GWAS/sequence data in PLINK/VCF; phenotype datasets in CSV/TSV
NIAGADS DSS [213]	AD/ADRD genomics	Whole-genome/exome data, including ADSP datasets; harmonized phenotypes; controlled-access cloud formats
ChEMBL [228]	Drug discovery	Bioactivity values (IC_50_, K_d_, K_i_); distributed in SQL/CSV
DrugBank [250]	Drug–target knowledge	XML, CSV, and SQL; also API-accessible

## Data Availability

The original contributions presented in this study are included in the article. Further inquiries can be directed to the corresponding author.
